# Assessing genetic counselors' teaching experience and approaches to teaching in the classroom

**DOI:** 10.1002/jgc4.70207

**Published:** 2026-04-13

**Authors:** Jessica Doxey, Bonnie J. Baty, Katharine Bisordi, Shannan Dixon

**Affiliations:** ^1^ Master's in Genetic Counseling Program University of Maryland School of Medicine Baltimore Maryland USA; ^2^ Department of Pediatrics University of Utah Salt Lake City Utah USA; ^3^ Department of Obstetrics, Gynecology, and Reproductive Services University of Maryland School of Medicine Baltimore Maryland USA; ^4^ Department of Pediatrics University of Maryland School of Medicine Baltimore Maryland USA

**Keywords:** classroom, education, genetic counseling, professional development, teaching

## Abstract

While most genetic counselors (GCs) are involved in teaching activities, there is little documentation of how GCs learn to teach and their responsibilities as educators. Past studies have focused on GCs' roles in teaching students in clinical settings as supervisors, but limited literature exists about GCs' roles as educators in classroom settings. This descriptive, exploratory study aimed to describe the current landscape of GCs as teachers in classroom settings and assess GCs' approaches to teaching in the classroom. GCs recruited from the National Society of Genetic Counselors, Maryland and DC Society of Genetic Counselors, and Genetic Counselor Educators Association completed an anonymous online survey that assessed classroom teaching experience, approaches to teaching using the Approaches to Teaching Inventory—Revised (ATI‐R), and training resources and methods they have used in the past and would like to use in the future to develop teaching skills. A total of 118 survey responses were analyzed using descriptive statistics, correlations, independent samples *t*‐tests, and analysis of variance (ANOVA). Participants reported teaching several different student populations, including genetic counseling students, medical students, residents, and fellows. The most common teaching activity GCs reported across all student populations was lecturing in genetics didactic courses. Higher scores on the conceptual change/student‐focused scale of the ATI‐R represented a more learner‐centered approach to teaching and were associated with a greater number of lectures taught (*p* = 0.028) and percentage of time devoted to teaching (*p* = 0.042). Participants infrequently had formal, degree‐based training in education and commonly reported that they would like to participate in continuing education unit activities and other workshops designed for education to further improve their teaching skills in the future. These findings may be used to inform the development of future training resources for GCs in their roles as classroom educators.

**What is known about this topic**

Genetic counselors play a critical role in educating genetic counseling students in classroom settings. However, most genetic counselors do not have formal training in education.

**What this paper adds to this topic**

This study illustrates genetic counselors' roles and approaches to teaching as classroom educators and highlights training resources that genetic counselors would like to use in the future to further improve teaching skills. Findings from this study may be used to inform the development of future training methods for genetic counselors in their roles as classroom educators.

## 1 INTRODUCTION

Educating others is a key responsibility of genetic counselors (GCs; Accreditation Council for Genetic Counseling, 2023). Not only do GCs educate patients in clinical settings but they also play a large role in the education and training of genetic counseling students in classroom settings (Gasparini et al., 2019). According to the 2023 National Society of Genetic Counselors Professional Status Survey (PSS), 86% of GCs report that they are involved in teaching activities to audiences including GC students, medical students, GCs, and physicians (National Society of Genetic Counselors, 2023). However, the PSS does not offer further insights into the teaching activities of GCs, such as the settings (i.e. clinical, classroom, community education) in which GCs are instructors or the teaching approaches of GCs. Furthermore, there is little documentation of how GCs learn to educate others in classroom settings. In order to inform the development of future training methods for GCs in their roles as classroom educators, it is imperative to understand GCs' current approaches to teaching and how they are trained to educate others.

### 1.1 GCs as educators

While GCs play an important role in educating GC trainees, there is a lack of research about GCs in their roles as educators. There is some literature that examines GCs' role in educating students in clinical settings as supervisors (Atzinger et al., 2016; Finley et al., 2016; Kennedy, 2000; McCarthy Veach, 2023), but to our knowledge, there is only one study that assesses GCs in their roles as teachers in classroom settings (Gasparini et al., 2019). In their 2019 study, Gasparini and colleagues surveyed GCs in the United States and Canada to examine GCs' perceived self‐efficacy as teachers. GCs who teach were asked to rate their perceived self‐efficacy on 30 items related to teaching competencies on a scale from 0 to 100, with higher scores representing higher self‐efficacy. Similar to a study examining the perceived self‐efficacy of GCs as supervisors (Finley et al., 2016), self‐efficacy scores for the teaching competency items were found to be high, with all items averaging higher than a score of 75 of 100.

Additionally, the study reported educational experience characteristics of GC teachers, such as years of experience teaching and training and resources that GCs have used in the past to develop and improve teaching skills (Gasparini et al., 2019). Informal methods of teaching training such as self‐reflection, trial and error and consultation with other GCs were each reported to have been used by over 90% of GCs. Conversely, GCs reported using formal training such as having a teaching certificate or degree in education (10.8%) or participating in an online (26.2%) or in person (34.6%) class/short course much less frequently than informal methods of training. Most GCs (82.3%) also reported that they would be interested in the development of teaching skills. More research is needed to further elucidate what training methods GCs have used in the past to develop teaching skills and what methods they would like to use in the future.

### 1.2 Faculty development programs and teaching approaches

In other health professions, it is also uncommon for educators to have formal, degree‐based teaching training (Swanwick, 2008). Starting in the late 19th century, medical educators have often been trained using Halsted's model of “see one, do one, teach one” rather than more formal methods of education (Kotsis & Chung, 2013). However, over the past 40 years, medical education programs have been shifting from using the Halsted model to using more formal methods of training such as faculty development programs (Burgess et al., 2019; Steinert, 2010; Swanwick, 2008). Such faculty development programs most commonly involve formal, structured activities in group settings and are designed to improve the performance of faculty members in teaching, research, and administration roles (Steinert et al., 2006, 2016).

Faculty development programs may focus on encouraging certain frameworks or teaching approaches to improve teaching skills. For example, a faculty development program may teach educators to organize educational goals through Bloom's taxonomy of cognitive gain to help students progress from more basic levels of learning like remembering information to higher levels of learning such as analyzing and evaluating information (Abdulghani et al., 2015; Krathwohl, 2002). Another goal of faculty development programs may be to encourage educators to adopt a learner‐centered approach (sometimes called a student‐focused approach) to teaching (Regan‐Smith et al., 2007; Steinert et al., 2016). Teachers who have a learner‐centered approach try to understand barriers that their students face in their education so that they can tailor their teaching to best fit the needs of their students (Srinivasan et al., 2011). Furthermore, teachers who take this approach actively monitor their students' understanding and aim to change their students' perceptions about subject matter (Trigwell et al., 2005). Learner‐centeredness has been associated with several positive outcomes, including students having a deeper approach to learning, higher class participation, and higher motivation to learn (Cornelius‐White, 2007; Trigwell et al., 1999).

### 1.3 Approaches to teaching inventory

In 1994, Trigwell, Prosser, and Taylor used a phenomenographic approach to describe qualitative differences in approaches to teaching of university physics teachers. This approach involves interviewing participants to identify and describe qualitatively different ways that participants understand and experience a phenomenon (Marton, 1981). After interviewing 24 university science teachers and analyzing the interview transcripts, the authors described a range of five different categories of approaches to teaching. The two most extreme categories, the information transmission/teacher‐focused approach and the conceptual change/student‐focused approach, were then used to create items for a preliminary, 16‐item version of the Approaches to Teaching Inventory (ATI), which included two scales: the Information Transfer/Teacher‐focused (ITTF) scale and the Conceptual Change/Student‐focused (CCSF) scale (Trigwell & Prosser, 1996). After further analysis of the ATI, Trigwell et al. (2005) developed a revised version, the Approaches to Teaching Inventory—Revised (ATI‐R), which includes 22 items.

The ITTF approach is described as an approach in which teachers focus on transmitting information to their students based on their own knowledge and engage in little or no interactions with their students (Trigwell et al., 1994, 2005; Trigwell & Prosser, 2004). Teachers who adopt this approach do not consider their students' prior knowledge to be important and assume that their students do not need to be an active part of the learning process (Trigwell & Prosser, 2004). Conversely, the CCSF approach is described as an approach in which educators aim to change students' conceptions of the topic they are studying (Trigwell et al., 1994, 2005; Trigwell & Prosser, 2004). Teachers who adopt a CCSF approach focus on students' existing knowledge and learning process and encourage students to construct their own knowledge of the topic (Trigwell & Prosser, 2004). Information transmission is also a part of the CCSF approach, but it is not thought to be sufficient for students' deeper learning (Trigwell et al., 2005). While the CCSF approach includes elements of the ITTF approach, the ITTF approach is more limited and does not include aspects of the CCSF approach (Trigwell et al., 2005). Therefore, the CCSF approach is described as a more complete approach to teaching than the ITTF approach (Trigwell et al., 2005).

The ATI has been used in several studies examining approaches to teaching, including a study about the relation between students' approaches to learning and teachers' approaches to teaching which showed that students with teachers who had an informational transmission approach to teaching were more likely to report that their approach to learning the subject matter was surface level (Trigwell et al., 1999). Conversely, students with teachers who had a more student‐focused approach to teaching that attempted to change students' conceptions were more likely to report that they had a deeper approach to learning (Trigwell et al., 1999). Surface level approaches to learning, which involve simply memorizing information, have been associated with poorer quality learning outcomes, while deeper approaches to learning, which involve attempting to make meaning of information and concepts, have been associated with higher quality learning outcomes (Trigwell & Prosser, 1991). Therefore, a teacher's approach to teaching may have important effects on student learning outcomes.

### 1.4 The current study

The overarching goal of the current study was to investigate GCs' roles as teachers and their approaches to teaching in the classroom. Specifically, this study aimed to (1) describe the current landscape of GC educators by identifying the teaching experience of GCs who are involved in classroom teaching, the student populations GCs are teaching, and the teaching activities that GCs engage in; (2) examine the training that GCs undergo to guide their classroom teaching practice and how they would like to continue to develop their teaching skills; (3) assess GCs' approaches to teaching in the classroom with the Approaches to Teaching Inventory—Revised (ATI‐R; Trigwell et al., 2005); and (4) evaluate whether GCs' approaches to teaching in the classroom are related to their teaching experience and the student populations they teach. We hypothesized that GCs with more classroom teaching experience utilize approaches to teaching that are more learner‐centered (represented by higher scores on the CCSF scale on the ATI‐R). Additionally, we hypothesized that GCs who only teach GC students have more learner‐centered approaches to teaching (represented by higher scores on the CCSF scale of the ATI‐R), but GCs who only teach non‐GC student populations utilize more teacher‐focused approaches to teaching (represented by higher scores on the ITTF scale on the ATI‐R).

## 2 METHODS

### 2.1 Participants

All GCs who were over the age of 18 and fluent in English were eligible to participate in this study. GCs were recruited from the National Society of Genetic Counselors (NSGC), Maryland and DC Society of Genetic Counselors (MDCGC), and Genetic Counselor Educators Association (GCEA).

### 2.2 Procedures

After approval from the University of Maryland, Baltimore Institutional Review Board (HP‐00106084), a link to the anonymous survey was sent to all members of the NSGC, MDCGC, and GCEA email listservs. The survey remained open for 3 months after the initial email was sent. A reminder email was sent to each email listserv approximately 2 weeks before the survey closed.

### 2.3 Instrumentation

Study participation involved an online survey administered through Qualtrics (see Appendix S1 for the full survey). The online survey was first piloted to a cohort of health profession education PhD candidates who all had many years of both classroom and clinical instruction experience, and minor wording changes were made to the survey. The final survey included four sections: (1) demographic information (5 items), (2) classroom teaching experience (22 items), (3) training methods and resources used by GCs to develop teaching skills (2 items), and (4) the ATI‐R (22 items). Demographic information collected included age, highest degree earned, years of experience as a GC, primary specialty area, and primary place of practice. Classroom teaching experience was explored using questions related to years of teaching experience, number of courses directed or co‐directed, number of lectures or class periods taught, percentage of work time devoted to teaching, student populations taught, number of students taught, and teaching activities used in the classroom with different student populations. Training methods and resources used by GCs to develop teaching skills were assessed by providing pre‐set categories as well as spaces for open‐ended responses. GCs were also asked to identify training methods and resources they would like to use in the future to improve their teaching skills.

The ATI‐R is a 22‐item inventory consisting of two scales: the Information Transfer/Teacher‐focused scale (ITTF) and the Conceptual Change/Student‐focused (CCSF) scale (Trigwell et al., 2005; see Appendix S1 for the full inventory). Each scale contains 11 items. Items for both scales are measured using a 5‐point scale, with 1 = “only rarely or never true,” 2 = “sometimes true,” 3 = “true about half the time,” 4 = “frequently true,” and 5 = “almost always or always true.” Both scales are scored by taking the mean numeric response of each item on the scale. Higher scores on the ITTF scale indicate a more teacher‐focused approach to teaching, and higher scores on the CCSF scale indicate a more student‐focused approach to teaching.

### 2.4 Data analysis

Descriptive and exploratory statistical analyses were performed using IBM SPSS Statistics, version 26.0 (IBM Corp., 2019). Descriptive statistics were calculated for demographic information, teaching experience, training and resources used by GCs, and ATI‐R scores for the descriptive analysis. Chi‐square tests were used to compare variables from this study with the 2023 PSS (National Society of Genetic Counselors, 2023). Correlations were used to compare ATI‐R scores and measures of teaching experience, including years of experience, total number of courses directed or co‐directed, total number of lectures taught, and percentage of work time devoted to teaching to test the exploratory hypothesis that GCs with more classroom teaching experience utilize approaches to teaching that are more learner‐centered (represented by higher scores on the CCSF scale on the ATI‐R). Additionally, independent samples *t*‐tests, analysis of variance (ANOVA) tests, Shapiro–Wilk tests, and Levene's tests were conducted to test the exploratory hypothesis that GCs who primarily teach GC students have more learner‐centered approaches to teaching (represented by higher scores on the CCSF scale of the ATI‐R), but GCs who teach other student populations utilize more teacher‐focused approaches to teaching (represented by higher scores on the ITTF scale on the ATI‐R).

## 3 RESULTS

A total of 127 survey responses were received. After excluding nine responses from analysis due to partial completion, the final study population consisted of 118 participants. Of the 118 responses used for analysis, two responses were only partially used for analysis due to answering the ATI‐R items based on clinical supervision experience or teaching experience during graduate school rather than classroom teaching experience as a GC. Since most members of MDCGC and GCEA are also members of NSGC, the response rate was calculated using NSGC membership, which included approximately 4500 members at the time the survey was sent, yielding a response rate of approximately 2.6%.

### 3.1 Demographics

Demographic variables of study participants are presented in Table 1. In terms of age, degrees earned, and years of experience as a GC, the sample in the current study was not significantly different than the sample in the 2023 PSS. The average age of participants in this study was 37.2 years (SD = 10.2). All participants (*n* = 118) had earned a master's degree, while three participants also had earned a PhD, one participant also had earned an MD, and five participants also had earned other degrees, such as another master's degree or a certificate in medical education. Participants had an average of 11.5 years (SD = 9.3) of experience as a GC.

**TABLE 1:** Demographics of study participants.

Variable	*n*	%
Age (*n* = 116)		
18–24	1	0.9
25–34	60	51.7
35–44	32	27.6
45–54	12	10.3
55+	11	9.5
Degree (*n* = 118)		
Master's degree	118	100.0
MD	1	0.8
PhD	3	2.5
EdD	0	0.0
JD	0	0.0
Other	5	4.2
Years of experience as a genetic counselor (*n* = 118)		
<1	2	1.7
1–4	30	25.4
5–9	32	27.1
10–14	16	13.6
15–19	15	12.7
20–24	7	5.9
25–29	5	4.2
30+	11	9.3
Primary specialty area (*n* = 118)		
Cancer genetics—adult	24	20.3
Cancer genetics—pediatric	1	0.8
Cardiology	3	2.5
Consumer genomics/personal genomics	1	0.8
General adult genetics	4	3.4
Genetic counselor training/education	21	17.8
Genomic medicine	1	0.8
Hematology	0	0.0
Laboratory sciences (molecular/cytogenetics/biochemical testing/variant science)	6	5.1
Metabolic disease	3	2.5
Neurogenetics	3	2.5
Newborn screening	1	0.8
Ophthalmology	0	0.0
Pediatrics	14	11.9
Pharmacogenetics	0	0.0
Preconception/reproductive screening	0	0.0
Preimplantation genetic testing, ART/IVF, infertility	1	0.8
Prenatal	24	20.3
Psychiatric	0	0.0
Public health	0	0.0
Other	5	4.2
No specific area of practice	6	5.1
Primary employer work setting (*n* = 118)		
Government organization or agency	4	3.4
Hospital/medical facility—academic medical center	58	49.2
Hospital/medical facility—private	4	3.4
Hospital/medical facility—public (including FQHC)	10	8.5
Insurance company/benefit management company	0	0.0
Laboratory—commercial	8	6.8
Laboratory—non‐commercial	4	3.4
Not‐for‐profit organization—other	1	0.8
Physician's private practice	0	0.0
Private company—biotechnology/research development	0	0.0
Private company—digital health/software	0	0.0
Private company—telegenetics/consulting/utilization management	1	0.8
Private company—other	0	0.0
Self‐employed/private practice	0	0.0
University, college, or training program	26	22.0
Other	2	1.7

*Note*: Study participants could select more than one item for degrees earned.

The current study used the same preset categories as the 2023 PSS to determine the primary specialty area and primary employer work setting of GCs. Although the most frequently reported primary specialty areas in both the current study and the 2023 PSS were adult cancer genetics and prenatal, the proportion of GCs in the current study who reported GC training/education as their primary specialty area (17.8%) was considerably higher than the proportion of the sample in the 2023 PSS who reported GC training/education as their primary specialty area (1.8%), *χ* ^2^ (1, *N* = 2835) = 120.12, *p* < 0.001. The most frequently reported primary employer work setting for both this study and the 2023 PSS was a hospital/medical facility at an academic medical center. However, the proportion of GCs in the current study who reported their primary employer work setting as a university, college, or training program (22.0%) was significantly higher than the sample from the 2023 PSS who reported their employer work setting as a university, college, or training program (3.5%), χ^2^ (1, *N* = 2954) = 95.03, *p* < 0.001.

### 3.2 Teaching experience

The study participants' teaching experience is presented in Table 2. On average, the participants had 6.8 years (SD = 7.2) of classroom teaching experience. Of note, 12.7% of participants (*n* = 15) reported having 0 years of teaching experience. The mean number of courses that participants had directed or co‐directed was 5.5 courses (SD = 6.9). However, 36.4% of participants (*n* = 43) reported that they have directed or co‐directed 0 courses. Most participants (95.7%) reported that they have taught at least one lecture or class period. The average number of lectures or class periods that participants reported teaching was 15.0 lectures (SD = 7.5), and 53.4% of participants (*n* = 63) reported teaching over 21 lectures or class periods. Slightly more than half of participants (*n* = 64; 54.2%) reported that they spend 1–10% of their time devoted to teaching.

**TABLE 2:** Teaching experience of study participants.

Variable	*n*	%
Years of classroom teaching experience (*n* = 118)		
0	15	12.7
<1	6	5.1
1–4	33	28.0
5–9	36	30.5
10–14	11	9.3
15–19	7	5.9
20–24	5	4.2
25–29	4	3.4
30+	1	0.8
Total number of courses directed or co‐directed (*n* = 118)		
0	43	36.4
1–5	36	30.5
6–10	14	11.9
11–15	10	8.5
16–20	5	4.2
21+	10	8.5
Total number of lectures/class periods taught (*n* = 118)		
0	5	4.2
1–5	17	14.4
6–10	15	12.7
11–15	14	11.9
16–20	4	3.4
21+	63	53.4
Percentage of time devoted to teaching (*n* = 118)		
0	6	5.1
1%–10%	64	54.2
11%–20%	15	12.7
21%–30%	5	4.2
31%–40%	8	6.8
41%–50%	4	3.4
51%–60%	9	7.6
61%–70%	3	2.5
71%–80%	3	2.5
>81%	1	0.8
Student populations taught by study participants (*n* = 114)		
Genetic counseling students	107	93.9
Medical students	70	61.4
Nursing students	22	19.3
Other health profession students	34	29.8
Undergraduate students	33	28.9
Residents or fellows	72	63.2
Other	13	11.4

Of the 114 participants that reported teaching different student populations, 93.9% (*n* = 107) reported teaching GC students, 63.2% (*n* = 72) reported teaching residents or fellows, 61.4% (*n* = 70) reported teaching medical students, 29.8% (*n* = 34) reported teaching other health profession students (e.g., students studying physical therapy, occupational therapy, speech therapy, pharmacy), 28.9% (*n* = 33) reported teaching undergraduate students, 19.3% (*n* = 22) reported teaching nursing students, and 11.4% (*n* = 13) reported teaching other student populations including other genetics master's or PhD students, law students, and kindergarten through grade 12 students. Table 3 shows teaching activities for different student populations and the average number of students taught at one time. Of note, the most common teaching activity that participants engaged in across all student populations was lecturing in genetics didactic courses.

**TABLE 3:** Teaching activities participants engaged in while teaching different student populations (*n* = 114).

Variable	GC^a^ students		Medical students		Nursing students		Other HP^b^ students		Residents or fellows		Undergraduate students		Other students	
	(*n* = 107)		(*n* = 70)		(*n* = 22)		(*n* = 34)		(*n* = 72)		(*n* = 33)		(*n* = 13)	
	*n*	%	*n*	%	*n*	%	*n*	%	*n*	%	*n*	%	*n*	%
Teaching activities														
Lectured in genetics didactic courses	99	92.5	55	78.6	19	86.4	28	82.4	56	77.8	23	69.7	9	69.2
Lectured in psychosocial didactic courses	39	36.4	5	7.1	1	4.5	1	2.9	6	8.3	5	15.2	0	0.0
Directed or co‐directed a course	69	64.5	4	5.7	1	4.5	3	8.8	5	6.9	4	12.1	3	23.1
Led team‐based activities	41	38.3	20	28.6	1	4.5	6	17.6	8	11.1	3	9.1	2	15.4
Led a workshop	40	37.4	22	31.4	0	0.0	9	26.5	9	12.5	4	12.1	3	23.1
Led a seminar	26	24.3	9	12.9	2	9.1	4	11.8	13	18.1	7	21.2	1	7.7
Led a journal club	21	19.6	6	8.6	1	4.5	0	0.0	5	6.9	1	3.0	0	0.0
Other	5	4.7	8	11.4	2	9.1	2	5.9	12	16.7	4	12.1	2	15.4
Average number of students taught at one time														
1–10	76	71.0	14	20.0	3	13.6	9	26.5	50	69.4	3	9.1	4	30.8
11–20	27	25.2	12	17.1	6	27.3	6	17.6	14	19.4	6	18.2	2	15.4
21–30	4	3.7	12	17.1	6	27.3	5	14.7	6	8.3	9	27.3	5	38.5
31–40	0	0.0	8	11.4	3	13.6	6	17.6	2	2.8	4	12.1	1	7.7
41–50	0	0.0	5	7.1	2	9.1	4	11.8	0	0.0	3	9.1	0	0.0
51+	0	0.0	18^c^	25.7	2	9.1	4	11.8	0	0.0	7	21.2	1	7.7

^a^ Genetic counseling students.

^b^ Other health profession students.

^c^ Of the 70 participants who reported teaching medical students, 11 (15.7%) participants reported teaching over 100 students at one time on average.

### 3.3 Teaching training resources and methods used by genetic counselors

**FIGURE 1:**
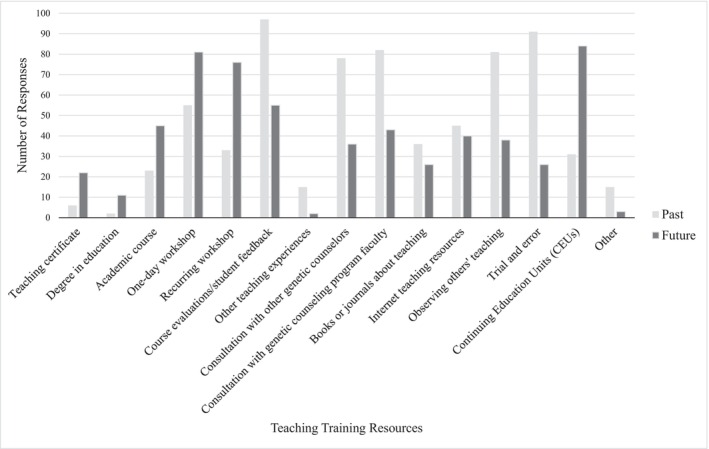
Teaching training resources that participants report using in the past and teaching training resources that participants would like to use in the future to develop teaching skills.

Participants were also asked to report training resources and methods they have used in the past to develop teaching skills as well as training resources and methods they would like to use in the future to improve their teaching skills (see Figure 1). The most frequently reported training resources included course evaluations/student feedback (82.9%), trial and error (77.8%), and consultation with GC program faculty (70.1%). The least commonly used training resources and methods identified by participants were a degree in education (1.7%), teaching certificate (5.1%), and other teaching experiences (12.8%). Fifteen participants (12.8%) reported that they have used training resources other than the resources specified, including teaching assistantships, consulting friends or family who are teachers, and formal observation and feedback from GC program leadership.

The most frequently selected training resources and methods that participants would like to use in the future included Continuing Education Units (CEU) through a professional organization (72.4%), 1‐day workshops (69.8%), and recurring workshops (65.5%). The least commonly selected resources that participants would like to use in the future included other teaching experiences (1.7%) and a degree in education (9.5%). Two participants (2.6%) reported that they would like to use resources other than the resources listed and specified that they would like to use teaching resources designed specifically for GCs.

### 3.4 ATI‐R

A total of 89 participants completed the ATI‐R. CCSF and ITTF scale scores were calculated for each participant by taking the mean of the 11 items on each scale. The lowest possible score for each scale was 1.0 and the highest possible score for each scale was 5.0. The mean score for the CCSF scale was 3.6 (SD = 0.7), with scores ranging from 1.7 to 5.0. The mean score for the ITTF scale was 3.3 (SD = 0.7), with scores ranging from 1.9 to 4.8.

Zero‐order correlations were performed between ATI‐R scale scores and measures of teaching experience, including years of experience, total number of courses directed or co‐directed, total number of lectures taught, and percentage of work time devoted to teaching (see Table 4). ITTF scores were not significantly related to any of the measures of teaching experience. CCSF scores were positively correlated with the total numbers of lectures taught (*r*(87) = 0.23, *p* = 0.028) and the percentage of time spent teaching (*r*(87) = 0.22, *p* = 0.042). Other measures of teaching experience were not found to be significantly related to CCSF scores. As expected, measures of teaching experience were correlated with each other.

**TABLE 4:** Zero‐order correlations of approaches to teaching inventory—revised scale scores and measures of teaching experience.

Variable	1	2	3	4	5	6	7	8
1. Information transfer/teacher‐focused (ITTF) scale	–							
2. Conceptual change/student‐focused (CCSF) scale	−0.30**	–						
3. Age in years	−0.09	0.14	–					
4. Years of experience as a genetic counselor	−0.14	0.11	0.95**	–				
5. Years of classroom teaching experience	−0.14	0.04	0.63**	0.72**	–			
6. Total number of courses directed or co‐directed	−0.18	0.13	0.39**	0.47**	0.50**	–		
7. Total number of lectures taught	−0.15	0.23*	0.52**	0.61**	0.57**	0.55**	–	
8. Percentage of time spent teaching	−0.10	0.22*	0.34**	0.32**	0.33**	0.63**	0.49**	–

*Note*: *p < 0.05; **p < 0.01.

Independent samples *t*‐tests showed that there was not a significant difference in the CCSF scores of participants who teach GC students (M = 3.6, SD = 0.7) and the CCSF scores of participants who do not teach GC students (M = 2.9, SD = 0.7); *t*(87) = −1.90, *p* = 0.06. However, GCs who teach GC students had higher ITTF scores (M = 3.4, SD = 0.7) than GCs who do not teach GC students (M = 2.5, SD = 0.5); *t*(87) = −2.64, *p* = 0.01.

Analysis of variance (ANOVA) tests were also conducted to test if GCs' ATI‐R scale scores differed between GCs who only teach GC students, GCs who only teach students who are not genetic counseling students, and GCs who teach both GC students and students in other fields of study. Data were tested for normality using Shapiro–Wilk tests and for homogeneity of variance using Levene's tests. The assumptions of normality and homogeneity of variance were met for each ANOVA. There was not a significant difference between the average CCSF scores between the three groups of GCs (*F*(30, 58) = 0.59, *p* = 0.94). However, there was a significant difference between the average ITTF scores between the three groups (*F*(2, 86) = 4.08, *p* = 0.02). Post hoc comparisons using the Tukey HSD test indicated that the mean ITTF score for GCs who only teach students who are not GC students (M = 2.5, SD = 0.5) was significantly different than the mean ITTF score for GCs who teach both GC students and students in other fields of study (M = 3.4, SD = 0.7). The mean ITTF score of the GCs who only teach GC students (M = 3.2, SD = 0.6) did not significantly differ from the other groups. These results suggest that ITTF scores were on average higher among participants who teach both GC students and other groups of students than participants who teach only non‐GC students.

## 4 DISCUSSION

The current study investigated the landscape of GC educators and their approaches to teaching in the classroom. This study illustrates GCs' important role as genetics educators for several different healthcare professionals and highlights training resources that GCs would like to use in the future to improve teaching skills, such as workshops and activities with CEUs. Findings from this study may be used to inform the development of teaching training methods for GCs. While the 2023 NSGC PSS found that 86% of GCs reported being involved in teaching activities in some manner, it did not provide further information about the teaching activities of GCs or differentiate between clinical teaching and classroom teaching (National Society of Genetic Counselors, 2023). The current study focused more specifically on GCs' experiences with classroom teaching and aimed to offer further insight regarding the teaching activities of GCs. GCs' classroom teaching experiences were also studied in Gasparini et al.'s (2019) study, which examined the teaching experience of GCs who teach GC graduate students. The educational experience characteristics of participants, such as the number of students taught, number of classes taught, and number of courses directed, in Gasparini and colleagues' study were similar to findings in the current study (Gasparini et al., 2019).

In addition to examining the classroom teaching experiences of GCs who teach GC students, the current study also investigated teaching activities that GCs engage in while teaching other student populations, such as medical students, undergraduate students, and residents and fellows. The most common teaching activity that GCs engage in across all student populations is lecturing in genetics didactic courses. As the demand for genetic services continues to increase with advancing technology and increased accessibility to genetic testing, GCs may play an important role in educating non‐genetics healthcare professionals to ensure that patients are receiving adequate care (Campion et al., 2019).

Training resources and methods that GCs have used in the past to develop teaching skills as well as resources that GCs would like to use in the future to improve their teaching were also examined in this study. Desired training differed considerably from previously accessed training. Similarly to what was found in Gasparini and colleagues' study in 2019, it was uncommon for participants in the current study to have formal teaching training such as a degree in education or a teaching certificate. Methods of training such as trial and error, consultation with GC program faculty, and course evaluations were more commonly used to inform teaching practice. Although GCs less commonly reported that they had previously attended 1‐day workshops, recurring workshops, or completed activities with Continuing Education Units (CEUs), these resources were the most highly reported training resources that GCs in this study would like to use in the future. While this study did not investigate the availability of workshops and CEU activities to help GCs develop teaching skills, this finding demonstrates that more opportunities for teaching development through workshops and other activities may be warranted.

GCs' approaches to teaching were also assessed in this study using the ATI‐R (Trigwell et al., 2005). The relationship between different measures of teaching experience and the ITTF and CCSF scales was examined to test the hypothesis that GCs with more classroom teaching experience utilize approaches to teaching that are more learner‐centered, represented by higher scores on the CCSF scale. This hypothesis was partially supported, as the total number of lectures taught and percentage of time spent teaching were found to be positively associated with CCSF scores. However, years of experience as a GC, years of classroom teaching experience, and the total number of courses directed or co‐directed were not found to be related to CCSF scores. Furthermore, ITTF scores were not found to be significantly related to any of the measures of teaching experience. These findings suggest that the number of lectures taught and percentage of time devoted to teaching may play a more important role in GCs' learner‐centeredness than their total years of experience.

GCs' approaches to teaching were also studied across different student populations. It was hypothesized that GCs who only teach GC students have more learner‐centered approaches to teaching (represented by higher CCSF scores), but GCs who teach only non‐GC student populations utilize more teacher‐focused approaches to teaching (represented by higher ITTF scores). This hypothesis was not supported by the current study, as there was not a significant difference between the CCSF scores of participants who teach GC students and those who do not teach GC students. Furthermore, a significant difference was found between the ITTF scores of participants who teach GC students and those who do not in the opposite direction as hypothesized. Participants who teach GC students were found to have higher ITTF scores than those who do not teach GC students. However, only 4/89 (~4%) participants reported that they only teach students who are not GC students. Therefore, the groups for comparison may not have been large enough to provide an accurate representation of differences in teaching approaches of GCs who teach different student populations. Larger sample sizes may be needed to accurately assess this hypothesis.

### 4.1 Study limitations

There are limitations of the study that may impact the interpretation of results. The ATI‐R measures participants' self‐perceptions of their approaches to teaching, which may not reflect their true approaches to teaching in practice. Student outcomes were not studied in relation to participants' approaches to teaching. Furthermore, no information was obtained about the amount of choice in the format and teaching strategies used. GCs invited to lecture in a course that is structured in a traditional didactic format may have less control over a learner‐centered approach than GCs encouraged to choose their own approach. Additional research is needed to determine if GCs' ATI‐R scores are related to student outcomes or the control they have over course or lecture format. The study also has limitations related to the sample of participants included in the study. The participants included a significantly higher proportion of GCs who primarily specialize in GC training/education and GCs who primarily work at a university, college, or training program than GCs who completed the 2023 PSS. Participants in the current study may be more interested in classroom education than the general population of GCs. The small sample size of GCs who do not teach GC students made it difficult to compare differences between the approaches to teaching of GCs who teach different student populations. Larger sample sizes including more GCs who only teach student populations other than GC students are needed to better study GCs' approaches to teaching across different student populations.

## 5 CONCLUSIONS

The current study was the first to assess GCs' approaches to teaching in the classroom. It addressed the gap in the literature by providing insights about GCs' classroom teaching experience, including teaching activities they engage in while teaching different student populations. As demonstrated by this study, GCs play an important role in the genetics education of many different healthcare professionals. It is imperative that GCs are prepared to teach both genetics and non‐genetics healthcare professionals to ensure that patients are receiving appropriate care. GCs in this study expressed an interest in participating in more workshops and activities with CEUs to further improve their teaching skills, which may be an important future direction for the development of trainings designed to help GCs develop effective teaching skills.

## AUTHOR CONTRIBUTIONS

Jessica Doxey contributed to the conception, study design, data collection, data analysis and interpretation, and drafting of the manuscript. Shannan Dixon contributed to the conception, study design, data collection, data interpretation, and manuscript revision. Bonnie J. Baty and Katharine Bisordi contributed to study design, data interpretation, and manuscript revision. Jessica Doxey confirms that she had full access to all the data in the study and takes responsibility for the integrity of the data and the accuracy of the data analysis. All of the authors gave final approval of this version to be published and agree to be accountable for all aspects of the work in ensuring that questions related to the accuracy or integrity of any part of the work are appropriately investigated and resolved.

## CONFLICT OF INTEREST STATEMENT

Jessica Doxey, Bonnie J. Baty, Katharine Bisordi, and Shannan Dixon declare that they have no conflict of interest.

## ETHICS STATEMENT

Human studies and informed consent: This study was reviewed and granted an exemption by the University of Maryland Baltimore Institutional Review Board. All procedures followed were in accordance with the ethical standards of the responsible committee on human experimentation (institutional and national) and with the Helsinki Declaration of 1975, as revised in 2000. Implied informed consent was obtained for individuals who voluntarily completed the online survey and submitted their responses.

Animal studies: No non‐human animal studies were carried out by the authors for this article.

## Supporting information


Appendix S1

## Data Availability

The data that support the findings of this study are available from the corresponding author upon reasonable request.
