# Supplementary material for: Assessing genetic counselors' teaching experience and approaches to teaching in the classroom
Source: J Genet Couns. 2026 Apr 13;35(2):e70207. doi: 10.1002/jgc4.70207 (PMC13074141; doi:10.1002/jgc4.70207)
Supplement: Supplementary file 1 — Appendix S1 [file JGC4-35-0-s001.docx]

**Supplemental Material**

## **Survey**

Start of Block: Demographics

What is your age in years?

▼ 20 ... 66+

What degree(s) have you earned? (select all that apply)

- Master's degree
- MD
- PhD
- EdD
- JD
- Other (please specify) __________________________________________________

How many years of experience do you have as a genetic counselor?

▼ 0 ... 31+ years

What is your current primary specialty area?

- Cancer Genetics - Adult
- Cancer Genetics - Pediatric
- Cardiology
- Consumer Genomics/Personal Genomics
- General Adult Genetics
- Genetic Counselor Training/Education
- Genomic Medicine
- Hematology
- Laboratory Sciences (Molecular/Cytogenetics/Biochemical Testing/Variant Science)
- Metabolic Disease
- Neurogenetics
- Newborn Screening
- Ophthalmology
- Pediatrics
- Preimplantation Genetic Testing, ART/IVF, Infertility
- Pharmacogenetics
- Preconception/Reproductive Screening
- Prenatal
- Psychiatric
- Public Health
- Other (specify) __________________________________________________
- I work without focus in a specific area of practice/I do not consider myself to have an area of practice

What is your current primary employer work setting? (In other words, what type of company are you employed by for your primary position?)

- Government Organization or Agency
- Hospital/Medical Facility - Academic Medical Center
- Hospital/Medical Facility - Private
- Hospital/Medical Facility - Public (including FQHC)
- Insurance Company/Benefit Management Company
- Laboratory - Commercial
- Laboratory - Non-commercial
- Not-For-Profit Organization - Other (please specify) __________________________________________________
- Physician's Private Practice
- Private Company - Biotechnology/Research Development
- Private Company - Digital Health/Software
- Private Company - Telegenetics/Consulting /Utilization Management
- Private Company - Other (please specify) __________________________________________________
- Self-employed/Private Practice
- University, College, or Training Program
- Other (please specify) __________________________________________________

End of Block: Demographics

Start of Block: Teaching Experience

How many years of classroom teaching experience do you have?

▼ 0 ... 31+ years

How many total courses have you directed or co-directed during your genetic counseling career?

For example, if you have directed or co-directed the same course for five years, your answer would be five.

▼ 0 ... 21+

How many total lectures/class periods do you estimate you have taught during your genetic counseling career?

For example, if you have taught the same lecture every year for three years, your answer would be three.

▼ 0 ... 21+

In the past year, what percentage of your work time has been devoted to teaching?

▼ 0% ... 91 - 100%

What student populations have you taught? (select all that apply)

- Genetic counseling students
- Medical students
- Nursing students
- Allied health (e.g., physical therapy, occupational therapy, etc.) students
- Undergraduate students
- Residents or fellows
- Other health profession students (e.g., dentists, pharmacists, etc.)
- Other (please specify) __________________________________________________

End of Block: Teaching Experience

Start of Block: Student Population: Genetic Counseling Students

Display This Question:

If What student populations have you taught? (select all that apply) = Genetic counseling students

You previously answered that you teach/have taught genetic counseling students. What teaching activities have you engaged in while teaching genetic counseling students? (select all that apply)

- Lectured in genetics didactic courses
- Lectured in psychosocial didactic courses
- Directed or co-directed a course
- Led team-based activities
- Led a workshop
- Led a seminar
- Led a journal club
- Other (please describe) __________________________________________________

Display This Question:

If What student populations have you taught? (select all that apply) = Genetic counseling students

On average, how many genetic counseling students do you teach at one time in the classroom?

▼ 0 ... 101+

End of Block: Student Population: Genetic Counseling Students

Start of Block: Student Population: Medical Students

Display This Question:

If What student populations have you taught? (select all that apply) = Medical students

You previously answered that you teach/have taught medical students. What teaching activities have you engaged in while teaching medical students? (select all that apply)

- Lectured in genetics didactic courses
- Lectured in psychosocial didactic courses
- Directed or co-directed a course
- Led team-based activities
- Led a workshop
- Led a seminar
- Led a journal club
- Other (please describe) __________________________________________________

Display This Question:

If What student populations have you taught? (select all that apply) = Medical students

On average, how many medical students do you teach at one time in the classroom?

▼ 0 ... 101+

End of Block: Student Population: Medical Students

Start of Block: Student Population: Nursing Students

Display This Question:

If What student populations have you taught? (select all that apply) = Nursing students

You previously answered that you teach/have taught nursing students. What teaching activities have you engaged in while teaching nursing students? (select all that apply)

- Lectured in genetics didactic courses
- Lectured in psychosocial didactic courses
- Directed or co-directed a course
- Led team-based activities
- Led a workshop
- Led a seminar
- Led a journal club
- Other (please describe) __________________________________________________

Display This Question:

If What student populations have you taught? (select all that apply) = Nursing students

On average, how many nursing students do you teach at one time in the classroom?

▼ 0 ... 101+

End of Block: Student Population: Nursing Students

Start of Block: Student Population: Allied Health Students

Display This Question:

If What student populations have you taught? (select all that apply) = Allied health (e.g., physical therapy, occupational therapy, etc.) students

You previously answered that you teach/have taught allied health (physical therapy, occupational therapy, etc.) students. What teaching activities have you engaged in while teaching allied health students? (select all that apply)

- Lectured in genetics didactic courses
- Lectured in psychosocial didactic courses
- Directed or co-directed a course
- Led team-based activities
- Led a workshop
- Led a seminar
- Led a journal club
- Other (please describe) __________________________________________________

Display This Question:

If What student populations have you taught? (select all that apply) = Allied health (e.g., physical therapy, occupational therapy, etc.) students

On average, how many allied health students (e.g., physical therapy, occupational therapy, etc.) do you teach at one time in the classroom?

▼ 0 ... 101+

End of Block: Student Population: Allied Health Students

Start of Block: Student Population: Undergraduate Students

Display This Question:

If What student populations have you taught? (select all that apply) = Undergraduate students

You previously answered that you teach/have taught undergraduate students. What teaching activities have you engaged in while teaching undergraduate students? (select all that apply)

- Lectured in genetics didactic courses
- Lectured in psychosocial didactic courses
- Directed or co-directed a course
- Led team-based activities
- Led a workshop
- Led a seminar
- Led a journal club
- Other (please describe) __________________________________________________

Display This Question:

If What student populations have you taught? (select all that apply) = Undergraduate students

On average, how many undergraduate students do you teach at one time in the classroom?

▼ 0 ... 101+

End of Block: Student Population: Undergraduate Students

Start of Block: Student Population: Residents or Fellows

Display This Question:

If What student populations have you taught? (select all that apply) = Residents or fellows

You previously answered that you teach/have taught residents or fellows. What teaching activities have you engaged in while teaching residents or fellows? (select all that apply)

- Lectured in genetics didactic courses
- Lectured in psychosocial didactic courses
- Directed or co-directed a course
- Led team-based activities
- Led a workshop
- Led a seminar
- Led a journal club
- Other (please describe) __________________________________________________

Display This Question:

If What student populations have you taught? (select all that apply) = Residents or fellows

On average, how many residents or fellows do you teach at one time in the classroom?

▼ 0 ... 101+

End of Block: Student Population: Residents or Fellows

Start of Block: Student Population: Other Health Professionals

Display This Question:

If What student populations have you taught? (select all that apply) = Other health profession students (e.g., dentists, pharmacists, etc.)

You previously answered that you teach/have taught other health profession students (e.g., dentists, pharmacists, etc.). What teaching activities have you engaged in while teaching other health professionals? (select all that apply)

- Lectured in genetics didactic courses
- Lectured in psychosocial didactic courses
- Directed or co-directed a course
- Led team-based activities
- Led a workshop
- Led a seminar
- Led a journal club
- Other (please describe) __________________________________________________

Display This Question:

If What student populations have you taught? (select all that apply) = Other health profession students (e.g., dentists, pharmacists, etc.)

On average, how many other health profession students (e.g., dentists, pharmacists, etc.) do you teach at one time in the classroom?

▼ 0 ... 101+

End of Block: Student Population: Other Health Professionals

Start of Block: Student Population: Other

Display This Question:

If What student populations have you taught? (select all that apply) = Other (please specify)

You previously answered that you teach/have taught another group of students that was not included in one of the provided categories of students. Please specify what other group of students you teach/have taught.

________________________________________________________________

Display This Question:

If What student populations have you taught? (select all that apply) = Other (please specify)

 What teaching activities have you engaged in while teaching the group of students you specified above? (select all that apply)

- Lectured in genetics didactic courses
- Lectured in psychosocial didactic courses
- Directed or co-directed a course
- Led team-based activities
- Led a workshop
- Led a seminar
- Led a journal club
- Other (please describe) __________________________________________________

Display This Question:

If What student populations have you taught? (select all that apply) = Other (please specify)

On average, how many of this group of students that you specified above do you teach at one time in the classroom?

▼ 0 ... 101+

End of Block: Student Population: Other

Start of Block: Training Methods and Resources

What training methods and resources have you used to develop teaching skills? (select all that apply)

- Teaching certificate
- Degree in education
- Academic course in teaching instruction as a part of an academic training program (e.g., genetic counseling program)
- Individual/standalone workshop, course, or training in teaching instruction attended in one day
- Recurring workshop or training in teaching instruction attended over multiple days outside of a general academic training program (e.g., professional development)
- Course evaluations/student feedback
- Other teaching experiences __________________________________________________
- Consultation with other genetic counselors
- Consultation with genetic counseling program faculty
- Books or journals about teaching
- Internet teaching resources
- Observing others' teaching
- Trial and error
- Continuing Education Units (CEU) through a professional organization (e.g., NSGC)
- Other __________________________________________________

What training methods and resources would you like to use in the future to develop teaching skills? (select all that apply)

- Teaching certificate
- Degree in education
- Academic course in teaching instruction as a part of an academic training program (e.g., genetic counseling program)
- Individual/standalone workshop, course, or training in teaching instruction attended in one day
- Recurring workshop or training in teaching instruction attended over multiple days outside of a general academic training program (e.g., professional development)
- Course evaluations/student feedback
- Other teaching experiences __________________________________________________
- Consultation with other genetic counselors
- Consultation with genetic counseling program faculty
- Books or journals about teaching
- Internet teaching resources
- Observing others' teaching
- Trial and error
- Continuing Education Units (CEU) through a professional organization (e.g., NSGC)
- Other __________________________________________________

End of Block: Training Methods and Resources

Start of Block: Approaches to Teaching Inventory - R

This inventory is designed to explore a dimension of the way that academics go about teaching in a specific context or subject or course. This may mean that your responses to these items in one context may be different to the responses you might make on your teaching in other contexts or subjects. For this reason, we ask you to describe your context.

Please name the subject/course of your response:

For each item please select one of the numbers (1-5). The numbers stand for the following responses:
 1 - this item was **only rarely or never** true for me in this subject.

2 - this item was **sometimes** true for me in this subject.
 3 - this item was true for me **about half the time** in this subject.

4 - this item was **frequently** true for me in this subject.
 5 - this item was **almost always or always** true for me in this subject.

**Please answer each item. Do not spend a long time on each: your first reaction is probably the best one.**

|  | 1  (only rarely or never true) | 2  (sometimes true) | 3  (true about half the time) | 4  (frequently true) | 5  (almost always or always true) |
| --- | --- | --- | --- | --- | --- |
| 1. In this subject students should focus their study on what I provide them. |  |  |  |  |  |
| 2. It is important that this subject should be completely described in terms of specific objectives that relate to formal assessment items. |  |  |  |  |  |
| 3. In my interactions with students in this subject I try to develop a conversation with them about the topics we are studying. |  |  |  |  |  |
| 4. It is important to present a lot of facts to students so that they know what they have to learn for this subject. |  |  |  |  |  |
| 5. I set aside some teaching time so that the students can discuss, among themselves, key concepts and ideas in this subject. |  |  |  |  |  |
| 6. In this subject I concentrate on covering the information that might be available from key texts and readings. |  |  |  |  |  |
| 7. I encourage students to restructure their existing knowledge in terms of the new way of thinking about the subject that they will develop. |  |  |  |  |  |
| 8. In teaching sessions for this subject, I deliberately provoke debate and discussion. |  |  |  |  |  |
| 9. I structure my teaching in this subject to help students to pass the formal assessment items. |  |  |  |  |  |
| 10. I think an important reason for running teaching sessions in this subject is to give students a good set of notes. |  |  |  |  |  |
| 11. In this subject, I provide the students with the information they will need to pass the formal assessments. |  |  |  |  |  |
| 12. I should know the answers to any questions that students may put to me during this subject. |  |  |  |  |  |
| 13. I make available opportunities for students in this subject to discuss their changing understanding of the subject. |  |  |  |  |  |
| 14. It is better for students in this subject to generate their own notes rather than copy mine. |  |  |  |  |  |
| 15. A lot of teaching time in this subject should be used to question students’ ideas. |  |  |  |  |  |
| 16. In this subject my teaching focuses on the good presentation of information to students. |  |  |  |  |  |
| 17. I see teaching as helping students develop new ways of thinking in this subject. |  |  |  |  |  |
| 18. In teaching this subject it is important for me to monitor students’ changed understanding of the subject matter. |  |  |  |  |  |
| 19. My teaching in this subject focuses on delivering what I know to the students. |  |  |  |  |  |
| 20. Teaching in this subject should help students question their own understanding of the subject matter. |  |  |  |  |  |
| 21. Teaching in this subject should include helping students find their own learning resources. |  |  |  |  |  |
| 22. I present material to enable students to build up an information base in this subject. |  |  |  |  |  |

End of Block: Approaches to Teaching Inventory - R
